# Supplementary figures and images for: Expressional and functional interactions of two Apis cerana cerana olfactory receptors
Source: PeerJ. 2018 Jun 11;6:e5005. doi: 10.7717/peerj.5005 (PMC6001824; doi:10.7717/peerj.5005)

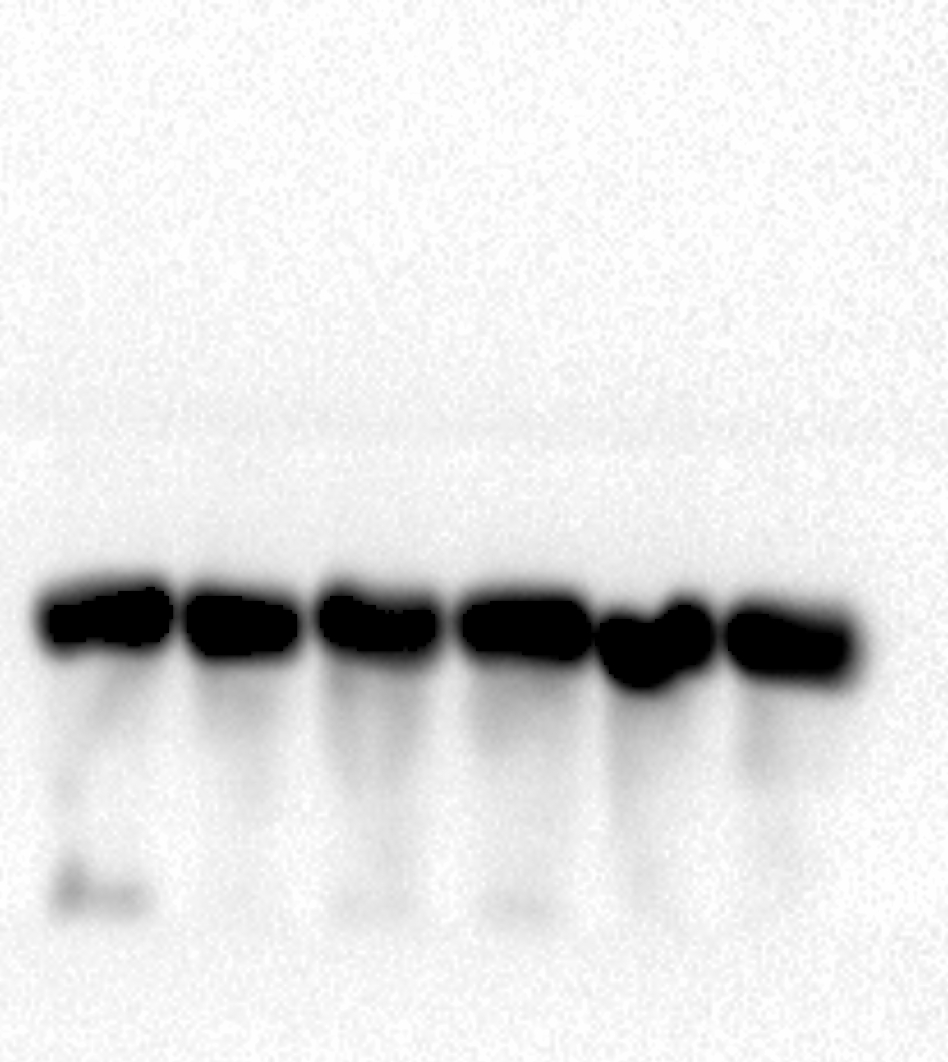

Supplement: Supplemental Information 1 — The expression of β-actin were determined by western blotting in Sf9 cells, pIB/V5-His transfected cells, recombinant His-tagged AcerOr1 transfected cells, and recombinant His-tagged AcerOr2 transfected cells. [file peerj-06-5005-s001.jpg]

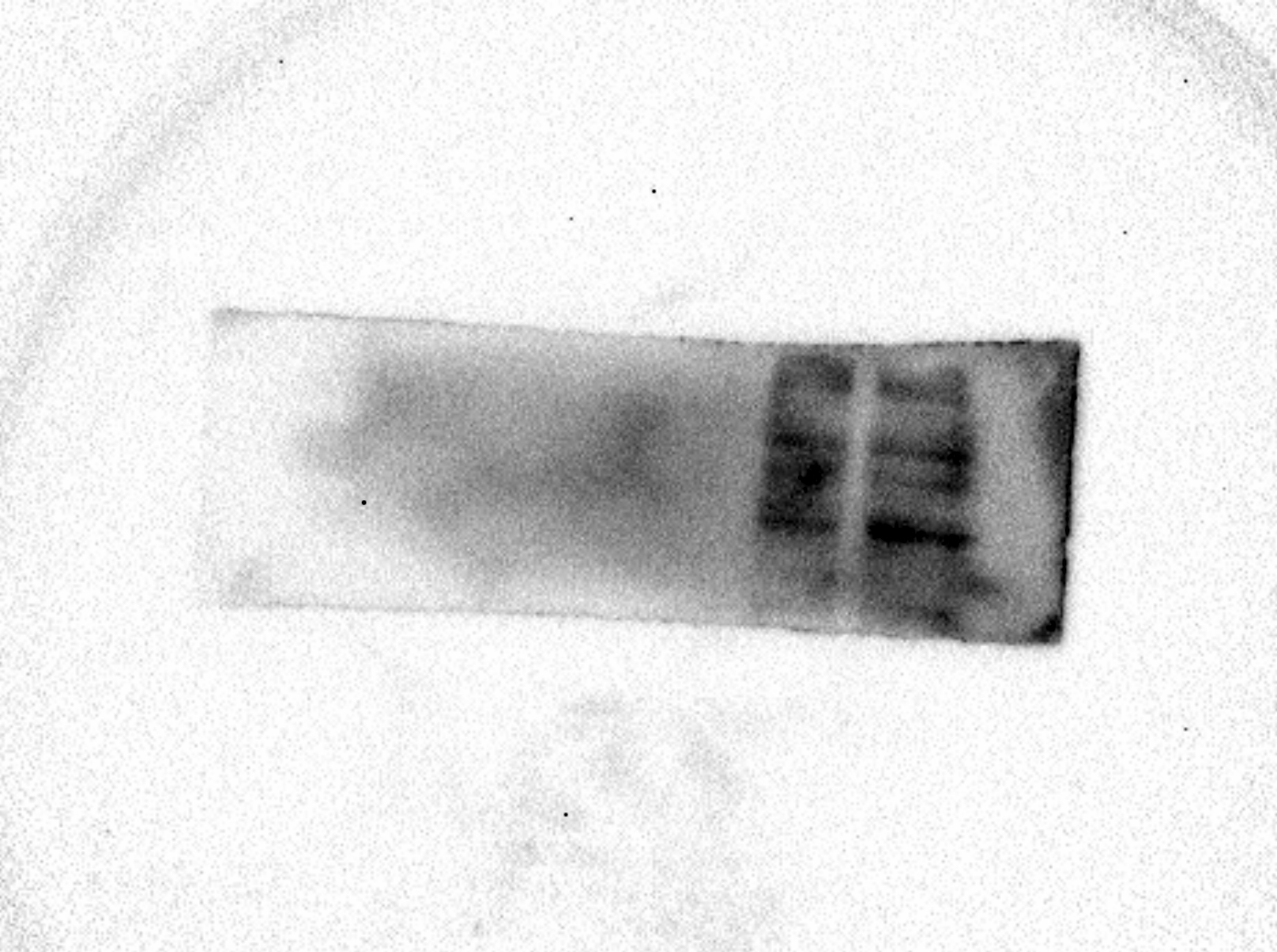

Supplement: Supplemental Information 2 — The expression of AcerOr2 were determined by western blotting in Sf9 cells, pIB/V5-His transfected cells, recombinant His-tagged AcerOr1 transfected cells, and recombinant His-tagged AcerOr2 transfected cells. [file peerj-06-5005-s002.jpg]

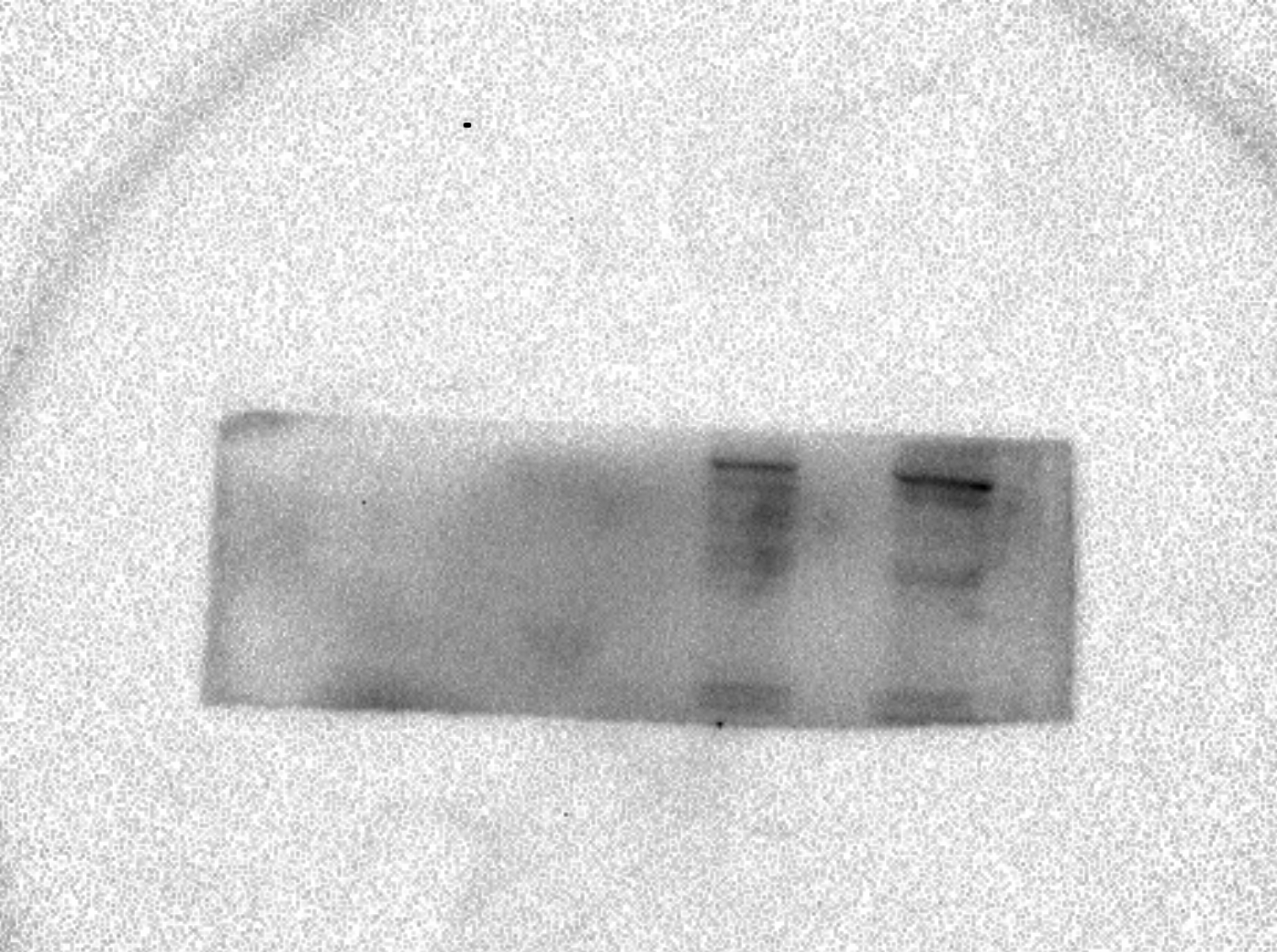

Supplement: Supplemental Information 3 — The expression of AcerOr1 were determined by western blotting in Sf9 cells, pIB/V5-His transfected cells, recombinant His-tagged AcerOr1 transfected cells, and recombinant His-tagged AcerOr2 transfected cells. [file peerj-06-5005-s003.jpg]
